# Cardiac protein changes in rats after soybean oil treatment: a proteomic study

**DOI:** 10.1186/s12944-015-0024-3

**Published:** 2015-04-14

**Authors:** Taisla Soprani, Vinicius Kuffer Uliana, Eduardo Hertel Ribeiro, Sergio Lisboa, Gabriella Xavier Maretto, André Teixeira Silva da Ferreira, Jonas Perales, Ivanita Stefanon, Suely Gomes de Figueiredo

**Affiliations:** 1 Pós-Graduação em Ciências Fisiológicas, Federal University of Espírito Santo (UFES), Av. Marechal Campos,1468, Maruípe, Vitória, Espírito Santo Brazil; 2 Laboratório de Toxinologia, Instituto Oswaldo Cruz, Fiocruz, Rio de Janeiro, RJ Brazil

**Keywords:** Comparative proteomic, Soybean oil, 2-DE, MS, Heart proteins

## Abstract

**Background:**

Several studies show that the consumption of vegetable oils, such as soybean oil, rich in polyunsaturated fatty acids (PUFAs) has beneficial health effects by preventing or reducing the risk factors of cardiovascular diseases. While the demonstration of beneficial effects of the consumption of unsaturated fatty acids on the cardiovascular system has been proven in a macroscopic level, the molecular/cellular mechanisms responsible for this phenomenon are poorly understood.

**Methods:**

In this work, a comparative proteomic approach, two-dimensional gel electrophoresis (2-DE) coupled to mass spectrometry (MALDI-TOF/TOF), was applied to investigate proteome differences in the left ventricle (LV) of rats that received 0.1 mL of soybean oil intramuscularly for 15 days (treated group - TR) and rats that had not (control group - CT).

**Results:**

Soybean oil treatment improved left ventricular function, TR animals presented lower value of LVEDP and significantly changed LV proteome. The protein profile of VE revealed differences in the expression of 60 protein spots (p < 0.05) between the experimental groups (CT and TR), 14 of those were identified by MS and MS/MS, and 12 of the 14 being non-redundant proteins. Robust changes were detected in proteins involved in cellular structure and antioxidant system and muscular contraction.

**Conclusions:**

The TR group presented an increase in the intensity of proteins involved in muscle contraction (myosin light chain-3 (3-MCL), creatine kinase M (CKM)) and thireodoxin, an antioxidant enzyme. Low intensity cytoskeletal protein, desmin, was also detected in TR animals. The results suggest that soybean oil induces changes in the levels of heart proteins which may partially account for the underlying mechanisms involved in the benefits provided by oils rich in polyunsaturated fatty acids.

**Electronic supplementary material:**

The online version of this article (doi:10.1186/s12944-015-0024-3) contains supplementary material, which is available to authorized users.

## Introduction

For over 50 years dietary guidelines have recommended consumption of low saturated fat and high complex carbohydrates to reduce the risk of cardiovascular disease. Despite these recommendations, a greater consumption of fructose, rapidly absorbed carbohydrates and a lower intake of complex carbohydrates and fat have been observed in industrialized countries [1],[2].

On the other hand, it has been demonstrated that increasing fat intake, which occurs mainly in diets composed of polyunsaturated fatty acids instead of carbohydrates, is beneficial in reducing coronary artery disease [2]-[5]. Dietetic supplementation with long-chain (C_16_ and C_18_) unsaturated fatty acids (PUFAs) has been shown to decrease insulin resistance, triglyceride levels, heart rate, blood pressure, and increase HDL cholesterol levels [6]. One important source of polyunsaturated fatty acids commonly used in industrialized countries is soybean oil. This oil is composed by triacylglycerols with a large proportion of unsaturated fatty acids, 54,5; 23,2 and 7,2% of linoleic (18:2, Δ^9,12^), oleic (18:1 Δ^9^) and linolenic (18:3, Δ^9,12,15^), respectively.

Linoleic acid has been associated with a reduction of fatal ventricular fibrillation in mice. A reduction in the incidence and severity of arrhythmias in ischemia after adding sunflower seed oil (rich in ω-6); and fish oil (rich in ω-3) to the diet of rats [7] was demonstrated. These suggest that the substitution of saturated fats for polyunsaturated fatty acids, ω-6 and ω-3, in the diet can reduce the likelihood of an ischemic event, avoiding sudden cardiac death [7].

Recently, Hertel Ribeiro and cols [8] demonstrated that the treatment of rats with soybean oil improved performance of the left ventricle without affecting blood pressure, and this was associated with an increase in the myosin ATPase activity and in the SERCA2a and sodium-calcium exchanger expression [8].

The purpose of the present study is to apply quantitative and comparative proteomics for the first time in examination of alterations in the LV proteomic profile of animals treated with soybean oil. The results may contribute to provide new insight into cellular mechanisms involved in the benefits provided by soybean oil.

## Material and methods

### Experimental design and animals

Eight-week old male wistar rats weighing 200-250 g each, were housed five per cage in a temperature-controlled environment (22-24°C) with a 12 h/12 h light–dark cycle and with free access to water and rat chow *ad libitum*. All procedures were approved by the local ethics committee of the Federal University of Espírito Santo State (protocol n° 052/2013).

The rats were randomized into two groups: control rats (CT – n = 8) and soybean oil treatment rats (TR – n = 8). The treated group received a daily dose of 0.1 mL (I.M.) of soybean oil for 15 days and the control group instead received a similar volume of 0.9% NaCl [8].

### Hemodynamic measurement

At the end of treatment, rats were weighed and anesthetized with urethane (1.2 g/kg; i.p.). A polyethylene catheter (PE50) filled with heparinized saline (50 U/mL) was introduced into their carotid artery to measure arterial systolic blood pressure (SBP) and diastolic blood pressure (DBP). The carotid artery catheter was introduced into the left ventricle to measure the systolic pressure (LVSP) and its positive and negative first derivatives (dP/dt max LV and dP/dt min LV, respectively), left ventricular end diastolic pressure (LVEDP), as well as heart rate (HR).

### Tissue sampling

After hemodynamic assessment, rats were sacrificed and their hearts were excised and washed with: Krebs-Henseleit solution to wash blood (four times); cold saline (three times); and cold Milli-Q water (twice). Then the atria and surrounding tissues were removed and the remaining mass of the heart (the ventricles) was separated into: left ventricle (LV) and right ventricle (RV). The tissue corresponding to the left ventricle was weighed and allocated in cryo tubes, which were immersed immediately in liquid nitrogen and kept at −80°C until use for proteomic analysis.

### Sample preparation

Four samples of LV from each experimental group were selected for randomized analysis, processed separately and used for the 2-DE gel analysis. LV protein extraction was performed as previously described [9] with some modifications. LV portions were pulverized in liquid nitrogen, then a portion (≅250 mg) of powdered LV was homogenized by an ultrasonic sonicator (XL-2000, QSonica) on ice in 10 volumes of two-dimensional lysis buffer (8 M urea, 2 M thiourea, 4% CHAPS, 40 mM Tris base and 0,2% protease inhibitor cocktail - Sigma-Aldrich). Then the homogenates were centrifuged (12.000 *g* for 45 min at 4°C) and the supernatants were collected and assayed for protein quantification (2-D Quant Kit – GE Healthcare). Aliquots of the protein extracts were separated into single-use samples and stored at −80°C until use.

### Two-dimensional gel electrophoresis (2-DE)

For the first dimension (isoelectric focusing), aliquots of 800 μg of LV soluble protein were diluted to a final volume of 350 μL in a rehydration buffer (Destreak, GE Healthcare) supplemented with 0.2% ampholytes pH 3–10. Samples were applied to the Immobiline DryStrips (17 cm; pH 3–10 NL; GE Healthcare) by gel rehydration. All isoelectric focusing was performed on a Protean IEF cell system (Bio-Rad) at a temperature of 20°C and a maximum current of 50 mA/strip. Focusing parameters were as follow: active rehydration (50 V) for 30 h; step 1 (300 V) for 3 h; step 2 (4.000 V) for 2 h; step 3 (4.000 V until complete 20.000Vh); step 4 (500 V) for 2 h. After the first-dimensional run, the IGP gel strip was incubated for 15 minutes in an equilibration buffer (50 mM Tris–HCl, pH 8.8; 6 M urea, 30% glycerol, 2% SDS, traces of bromophenol blue) containing 10 mg/mL dithiothreitol, followed by a second incubation in the same equilibration buffer containing 25 mg/mL iodoacetamide for 15 min. Then the IGP strips were sealed using 0.5% agarose in standard Tris-Glycine-SDS electrophoresis buffer. The second dimension electrophoresis was performed with 12.5% polyacrylamide gels [10] in Ettan DALTsix eletrophoresis unit (GE *Healthcare life sciences)* at 40 mA/gel until the dye front reached the bottom of the gel at 15°C. Gels were stained with colloidal Coomassie Blue Brilliant G-250, [11].

### 2-DE image analysis

Gel images were documented using an ImageScanner III calibrated densitometer (GE Healthcare). Eight two dimensional gels were obtained, four independent samples (4 biological replicates) per group. Data from 2-DE gels were analyzed using the Image Master 2D Platinum software 7.05 (GE Healthcare). The authenticity and outline of each spot were validated by visual inspection and edited when necessary. The intensity of each protein spot was normalized relative to the total abundance of all valid spots. Spots that exhibited significant differences between the groups (CT and TR) were selected for protein identification by mass spectrometry. Significance of differences observed was determined by the analysis of variance (ANOVA), and α < 0.05 was adopted as the level of significance.

### Identification of spots by mass spectrometry

Protein spots with differential intensity were manually excised from the gel. Gel pieces were then distained by rinsing three times of 15 minutes with 50% acetonitrile in 25 mM ammonium bicarbonate, followed by pure acetonitrile washing for five minutes, after being dried by SpeedVac (Eppendorf). Dried gel fragments were then rehydrated with 10 μL of protease solution (Trypsin Gold, Mass Spectrometry Grade, Promega, at 20 ng/μL in 50 mM ammonium bicarbonate) for 30 min in ice, then 20 μL of 50 mM ammonium bicarbonate was added and digestion was carried out for 16 h at 37°C. The peptides formed by digestion were extracted from the gel by washing twice, over constant shaking for 30 minutes each, with 30 μL of 50% acetonitrile/5% formic acid. Trypsin digests were concentrated in a SpeedVac (Eppendorf) to about 10 μL. The protein digests were subjected to a desalting step using Zip-Tip (C18 resin; P10, Millipore) as previously reported [12].

The tryptic peptides were analysed with a MALDI-TOF–TOF AB Sciex 5800 (AB Sciex, Foster City, CA) mass spectrometer. MS and MS/MS spectra were acquired in reflector mode to ensure optimal mass accuracy and peak resolution. Usually up to 15 of the most intense ion signals with signal to noise ratios above 2 were selected as precursors for MS/MS acquisition. During this data dependent analysis, an exclusion list with common trypsin autolysis masses and keratine masses was applied. External calibration in MS mode was performed using a mixture of five peptides: des-Arg1-Bradykinin (m/z = 904.468), angiotensin I (m/z = 1296.685), Glu1-fibrinopeptide B (m/z = 1570.677), and ACTH (18–39 clip) (m/z = 2465.199), and ACTH (7–38 clip) (m/z = 3657.929). Similarly, tandem mass spectra were externally calibrated using known fragment ion masses observed in the MS/MS spectrum of Glu1-fibrinopeptide B.

MS and MS/MS spectra were combined by BioTools (BrukerDaltonics) and used for search against the NCBI nonredundant database using the MASCOT® software (http://www.matrixscience.com). Search parameters were as follows: no restrictions on protein molecular weight; *Rattus* taxonomic; only tryptic peptides with one missed cleavage were considered; fixed and variable modifications were carbamidomethylation of Cys residues and Met-oxidation, respectively; mass accuracy of 0.8 Da were acceptable for matching peptides (MS and MS/MS mode).

Gel fragments with no protein and gel fragments from the molecular weight standard (bovine albumin) were used as negative and positive controls, respectively. Protein identified by homology with *Rattus novergicus* species, and with a global Mascot score with α < 0.05 was considered significant. Biological processes categorization was based upon information provided by Gene Ontology (GO); PANTHER [13] and UniProt’s database classification system (for *Rattus norvegicus*).

### Statistical analysis

Results are presented as mean ± SEM. Values were analysed by GraphPad Prism version 5.0 using an unpaired Student’s *t*-test, α < 0.05 was adopted as the level of significance.

## Results

### General characteristics and cardiac hemodynamic

At the end of the treatment, the body weight of animals treated with soybean oil was similar to the body weight of animals in the control group (CT: 310 ± 11.6 vs. TR: 301 ± 9.28 g). Soybean oil treatment did not cause significant alterations in the left ventricle to body weight ratio compared to controls (CT: 0.62 ± 0.04 vs. TR: 0.71 ± 0.04 mg/g), suggesting that ventricular hypertrophy did not occur.

No significant differences were found in heart rate, systolic blood pressure, diastolic blood pressure, left ventricular systolic pressure, maximal rate of pressure development; or maximal rate of pressure decay between groups (Table 1). However the TR animals showed a lower LVEDP (29.2%) when compared to controls.

**Table 1:** **Hemodynamic measures from control (CT) and soybean oil-treated rats (TR)**

	CT (n = 8)	TR (n = 8)
**HR,** bpm	374 ± 18	342 ± 14
**SBP,** mmHg	114.9 ± 4.6	102.57 ± 4.88
**DBP,** mmHg	81.6 ± 5.9	73.75 ± 5.44
**PAM,** mmHg	98.8 ± 4.9	82.5 ± 6.08
**LVSP,** mmHg	128.4 ± 6.6	118.8 ± 5.07
**LVEDP,** mmHg	6.08 ± 0.41	4.31 ± 0.26*
**dP/dt**_**+,**_mmHg/s	6656. ± 691	6066 ± 434
**dP/dt**_**-,**_mmHg/s	8061 ± 397	6994 ± 414

HR - heart rate; SBP - systolic blood pressure; DBP - diastolic blood pressure; LVSP left ventricle systolic pressure; LVEDP - left ventricle end diastolic pressure; dP/dt + maximal rate of pressure development; dP/dt _−_ maximal rate of pressure decay. Data are mean ± S.E.M. Student “t” test, *p < 0.05 vs. CT.

### Comparative proteome analysis of the left ventricle of animals control and treated with soybean oil

In order to evaluate the effect of soybean oil on proteins of LV, a total of eight (four per group) large format 2-DE gels were obtained, and used for differential proteome analysis. An outline of the gel figures are shown in the Additional file 1. The Factor analysis already suggests differences in the protein profile of the left ventricle in the CT and the TR groups. An outline of the factor analysis is shown in the Additional file 2. The Coomassie blue staining spot pattern was observed to be comparable between groups.

The image analysis of 2-DE gels detected an average of 401 spots for the CT group and 367 for the TR group. The 2-DE protein profiles obtained from CT and TR groups were highly reproducible concerning total number of protein spots, intensities and relative positions within the same group (correlation coefficient >0.94).

**Figure 1:**
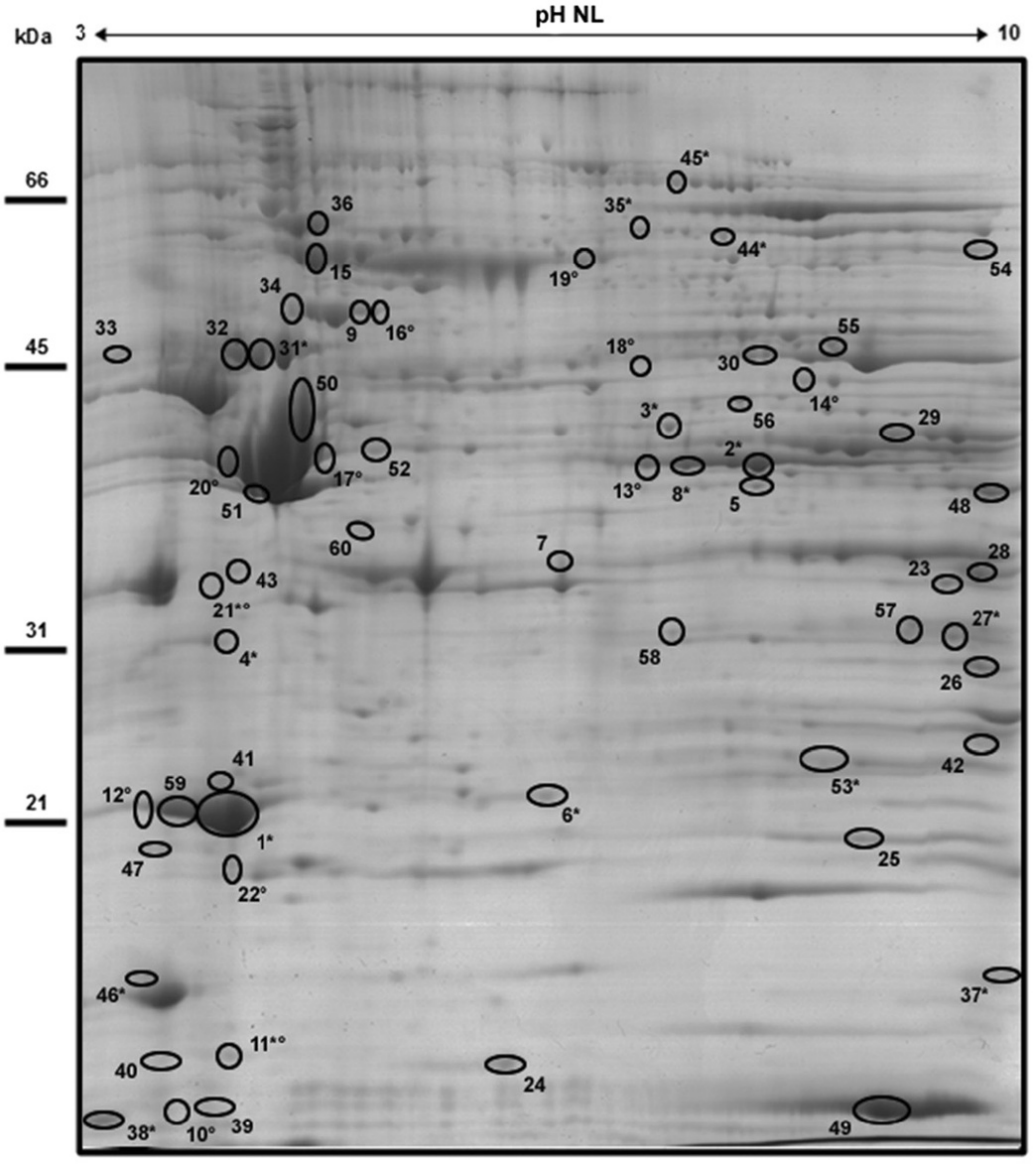
Representative 2-DE protein extracts of the left ventricle of the an animal CT. The numbered and surrounded points are statistically different (p <0.05) between groups. (°) spots only visualized in the gels of the TR group. (*) proteins identified by MS and MS/MS.

Analysis was performed by the Image Master 2D Platinun v 7.05 software analysis and indicated that 60 proteins spots was significantly different (p < 0.05) between CT and TR group. Out of the 60 spots 39 (65%) were down-regulated, and 21 (35%) were up-regulated in the TR group compared to the CT group. A representative CT 2-DE gel is shown in Figure 1, in which numbers indicate significantly different spots between analyzed groups.

### Protein identification of differentially expressed protein spots

Of the 60 differentially expressed protein spots, 39 were removed from the gels and processed for analysis by mass spectrometry system for MALDI/TOF-TOF.

The remaining spots were clustered and indistinguishable to the naked eye, therefore were not used for analysis in MALDI/TOF-TOF. Protein identification by MS/MS was successfully obtained for 14 spots. The same gene product was identified in two different spots, indicating potential diversity of protein forms including post-translational modifications (PTMs), mutations and/or isoforms. Thus, the 14 identified gel spots comprise 12 gene products that were differentially expressed between CT and TR left ventricular tissue. Proteins identified are indicated in Figure 1 by asterisk and listed in Table 2.

**Table 2:** **Proteins expressed differently in CT and TR groups**

Spot^a^	Protein name (ID)	Variation of spot protein intensity in LV TR group	Biological process^b^	Identified peptide sequences	Mascot – score proteico^c^	pI (teórico)	MM kDa (teórico)
**1**	Myosin light chain-3 (**P09542**)	↑	Muscle contraction	ITYGQCGDVLR	96	5,03	22,2
**2**	Creatine kinase M-type (**P00564)**	↑	Phosphocreatine biosynthetic process	GYTLPPHCSR;	65		
				DLFDPIIQDR;	91		
				LGSSEVEQVQLVVDGVK;	143		
				TGTAEMSSILEER;	62		
				FEEILTR;	51	6,58	43,2
				GGDDLDPNYVLSSR;	85		
				SFLVWVNEEDHLR;	126		
				RGTGGVDTAAVGAVFDISNADR	117		
**3**	Tu translation elongation factor, mitochondrial (predicted), isoform (**P49411**)	↑	Protein biosynthesis	AEAGDNLGALVR;	82		
				YEEIDNAPEER;	84		
				ADAVQDSEMVELVELEIR;	133		
				KYEEIDNAPEER;	104	7,23	44,0
				LLDAVDTYIPVPTR;	113		
				GITINAAHVEYSTAAR;	137		
				GEETPVIVGSALCALEQR;	176		
				DLEKPFLLPVESVYSIPGR	191		
**4**	Myosin light chain (**P09542**)	↑	Muscle contraction	HVLATLGER;	72		
				NKDTGTYEDFVEGLR;	124		
				AAPAPAAAPAAAPEPERPK;	130	5,03	22,2
				HVLATLGER;	72		
				NKDTGTYEDFVEGLR;	124		
				AAPAPAAAPAAAPEPERPK	130		
**6**	Thioredoxin-dependent peroxide reductase (**Q9Z0V6**)	↓	Antioxidant	SVEETLR	47	7,15	28,3
**8**	Creatine kinase M-type (**P00564**)	↓	Phosphocreatine biosynthetic process	GTGGVDTAAVGAVFDISNADR	231	6,58	43,2
**21**	Preprohaptoglobin (**P06866**)	↑	Acute phase Immunity	GSFPWQAK;	43		
				MGYVSGWGR;	52		
				YVMLPVADQEK;	48	6,10*	30,4
				SCAVAEYGVYVR	64		
**26**	Vdac 1 protein, partial (**Q9Z2L0**)	↓	Apoptosis/Ion transport	GYGFGLIK;	72		
				LETAVNLAWTAGNSNTR;	98		
				KLETAVNLAWTAGNSNTR;	167		
				VNNSSLIGLGYTQTLKPGIK;	143	8,55	30,8
				EHINLGCDVDFDIAGPSIR;	207		
				WNTDNTLGTEITVEDQLAR;	189		
				TDEFQLHTNVNDGTEFGGSIYQK	217		
**31**	Desmin (**P48675**)	↓	Muscle protein	LQEEIQLR;	65	5,21	53,4
				RIESLNEEIAFLK	66		
**37**	NADH desidrogenase [ubiquinone] (**Q91VD9**)	↓	Electron transport/Respiratory chain	HGWSYDVEGR;	90		
				SYGANFSWNKR;	33	10,14	19,7
				LDVTPLTGVPEEHIK	82		
**44**	Long-chain-fatty-acid-CoA ligase 1 (**P18163**)	↓	Fatty acid metabolism	VLKPTIFPVVPR	56	5,67	76,3
**45**	Glycogen phosphorylase, muscle form (**P09812**)	↓	Carbohydrate metabolic process	DYYFALAHTVR	98	6,91	97,7
**46**	chaperone activity of bc1 complex-like, mitochondrial (**Q5BJQ0**)	↓	Ubiquinone biosynthesis	EAGLSGQATSPLGR;	109	98	72,7
				EGPAPAYVSSGPFR	130		
**53**	ES1 protein homolog, mitochondrial precursor (**P56571**)	↓	-	ITNLAQLSAANHDAAIFPGGFGAAK	98	9,11	28,4

^a^ Spot ID - The numbers correspond to the specific spots as indicated in Figure 1. ^b^Functional categories according to Gene Ontology, Panther and UniProt biological process annotations. ^c^ With statistical significance (p <0.05) in protein homology and identity. ^*^pI related to the protein, it has not been found the pI of prepropeptide.

The identified proteins fell into several functional categories and subcellular localization. The classification of these proteins using the subcellular localization database revealed that the differentially expressed proteins comprised cytoplasmic, mitochondrial, cytoskeletal and myosin-complex related proteins. GO- and UniProt-based biological process categorisation showed these proteins to be involved in muscle contraction and cellular structure, energy production (lipids, carbohydrates metabolism and proteins of electron transport chain of oxidative phosphorylation) and antioxidant proteins. Notably, the TR group showed up-regulation of contractile proteins, such as the myosin light chain-3 and creatine kinase M-type and antioxidants, such as thioredoxin. Structural proteins, as desmin, showed downregulation.

## Discussion

An increasing amount of data seems to imply that polyunsaturated fatty acids are important in cardiovascular disease risk reduction both in the general population and in patients with preexisting heart disease. Evidence shows that supplementation with ω-3 at doses >3 g/day can lessen cardiovascular disease risk factors, including plasma triacylglycerols, blood pressure, platelet aggregation, and inflammation, while improving vascular reactivity [14].

The left ventricular isovolumetric systolic pressure and inotropic response to extracellular Ca^2+^ and isoproterenol were higher in the soybean-treated animals than in the control group. These changes were associated with increased myosin ATPase activity and alterations in calcium handling proteins, which are related to positive inotropism and regulation and maintenance of sarcoplasmic reticulum calcium load [15],[16].

Considering that vegetable oils could promote cardiac benefits by inducing changes in the heart proteins levels. In the present study a proteomic approach was used to demonstrate the differences between the CT group LV proteome profile and the TR group with soybean oil injected intramuscularly for 15 days LV proteome profile. Tissue samples were analyzed by 2-DE-MS/MS and resulted in the identification of proteins that may be involved in the significant improvement of ventricular function after the soybean treatment.

Proteome comparison between CT and TR identified 60 intensity changes in protein spots, 14 (23.3%) of which were identified by MS/MS and account for 12 non-redundant proteins. The protein changes identified could be categorized into 6 classes, using GO- and UniProt-base, according to their functional significance: (i) proteins of cardiac muscle contraction: myosin light chain-3 (MCL-3); creatine kinase M-type (CKMM); (ii) energy metabolism: NADH dehydrogenase, glycogen phosphorylase, long-chain-fatty-acid-CoA ligase 1; (iii) Structural: desmin; (iv) stress: bc1 chaperone, thioredoxin, preprohaptoglobin; (v) protein membrane: Vdac 1; (vi) of protein biosynthesis: elongation factor Tu.

Myosin light chain proteins are essential components in the generation of actin-based contractile force. As reported by our analysis, two protein spots (1 and 4) corresponding to myosin light chain-3 were up-regulated 1.33 and 3.24 fold respectively in the treated group. This data is in accordance with several studies that demonstrate that lowering the expression of myosin is associated with contractile dysfunction observed in dilated cardiomyopathy [17] and heart failure [18].

In addition we observed a large decrease of desmin, a muscle-specific intermediate filament protein type III, in the TR animals. This result is consonant with numerous independent studies that demonstrate involvement of desmin in human and experimental animal cardiac diseases comprehending accumulation of desmin deposits [19].

Faber and employees, performing a proteomic analysis of the RV of rats presenting pathological hypertrophy, observed increased desmin expression in this scenario [20]. Another study involving human hearts suffering from congestive heart failure identified, by western blot, an increase of desmin protein expression [19]. Also, increases in the protein expression level of this intermediate filament have also been observed in a hypertrophic cardiomyopathy (HCM) mouse model [21]. It was suggested that desmin overexpression in HCM mice is a compensatory or adaptive response to cell integrity loss in an attempt to reinforce the contractile units of the myocyte, thereby enhancing contractility [20]. Desmin has proven to be a sensitive biomarker for evaluating the deterioration degree of myocardial infarction [22] and it was designated as an intracellular marker for heart failure [23],[24]. We speculate that both the increase in intensity of myosin light chain-3 and the decrease in desmin levels may be associated with the improved cardiac function observed in the oil treated group.

Moreover, pronounced changes in creatine kinase type M (CK-M), which belongs to the category of cardiac muscle contraction proteins, was observed in this proteomic study. Creatine kinase (M-type) is a key intracellular enzyme that provides temporal and spatial energy buffering. It catalyses the reversible transfer of a phosphate moiety between creatine and ATP and it plays a central role in supplying energy to several tissues, such as skeletal muscle tissue and heart tissue. CK deficiency is a hallmark of cardiovascular diseases [25], suggesting that it is essential to myocardial energy homeostasis. Spindler and cols. showed that creatine kinase-deficient hearts exhibit increased susceptibility to ischemia-reperfusion injury and impaired calcium homeostasis [26].

In this work CK-M was identified in two distinct protein spots (2 and 8). It may be suggested that the acid form is phosphorylated and hence a post-translational modification of the basic form (PTM). However, an imbalance was found in the intensity levels of these spots. The CK more basic form (spot 2) and its less basic form (spot 8) exhibited higher and lower intensity in the treated group, repectively. Despite this imbalance, there is an increase in absolute abundance of this enzyme in treated oil animals (1.27 fold). Because a proportion of M type-CK is associated with sites of excitation-contraction coupling providing energy for contractility and SR-Ca^2+^ reuptake [27],[28], the over-regulation of CK, found in treated animals in this work, may be one possible explanation for the improved performance of the LV induced by unsaturated oils, which is consistent with cardioprotection afforded by unsaturated oils.

A large body of research has suggested that highly reactive oxygen derived free radicals (ROS) of endogenous or environmental origin play a cognitive role in the genesis and progression of various CVDs [29],[30]. In this work, expression pattern modification in two antioxidant enzymes was found. There was an increased intensity of the two in the treated group. Thioredoxin - Trx (1.68 fold) and haptoglobin - Hp were identified only in the TR group. Txr protects radical-sensitive enzymes from oxidative damage using a radical-generating system. It acts synergistically with MAP3K13 to regulate the activation of NF-kappa-B in the cytosol. Haptoglobin also acts as an acute phase protein and has been associated with a variety of common disorders (e.g. cardiovascular disease, autoimmune disorders, malignancy) [31].

In agreement with our findings, the plasma low levels of Hp have been associated with a worse functional outcome after myocardial infarction [32]. In addition, Tuncay and colleagues [33] suggested the intake of ω-3 has an important beneficial effect in the protection of diabetes-induced cardiac dysfunction. They also showed the levels of Trx reductase and other enzymes that exhibit antioxidant activities decrease in the diabetic heart, a situation that was normalized after treatment with omega-3, similar to the results in this study.

Although previous studies have reported the over expression of calcium handling proteins (sodium-calcium exchange - NCX and SERCA2a) [8], we have not identified ion transport proteins in this study, with the exception of the Vdac I (more abundant in CT group). Vdac forms a channel through the mitochondrial outer membrane and also through the plasma membrane.

Besides its ion transport and porine functions, Vdac has also been shown to play a role in the apoptotic process [34] and a key role in regulating metabolic and energetic flux across the outer mitochondrial membrane. The speculative higher levels of Vdac in the CT group seem consistent with the key role the molecule has in coupling glycolysis to oxidative phosphorylation [35] this could also be associated with the higher levels of glicogen phosphorylase in CT animals. On the other hand, we cannot associate the other differentially expressed proteins with cardiovascular improvement induced by soybean oil.

Despite the advantages of applying quantitative proteomics to examine the effects of soybean oil on LV rats proteome, there are limitations that must be considered, (i) low protein resolution in the gel basic area, (ii) the protein extraction method was not efficient for membrane proteins; (iii) identification of only 23.3% of protein spots with differences in intensity. A more sensitive method (e.g. Ettan DIGE) could be used for great proteomic hits.

In conclusion, we have demonstrated for the first time that soybean oil induces alterations in the rats’ LV proteome. Our study revealed that alterations in the expression level of proteins involved in muscle contraction, cellular structure and antioxidant system in LV treated animals can be correlated with improvement in left ventricular performance. These data corroborate the understanding of the mechanisms involved in cardioprotection afforded by this specific oil.

## Additional files

## Electronic supplementary material

Additional file 1:2-DE gels images obtained from four independent samples (4 biological replicates) per group (CT and TR).(DOC 2 MB)

Additional file 2:Graphic of projection of the gels obtained by factor analysis.(DOC 34 KB)

## Authors’ original submitted files for images

Below are the links to the authors’ original submitted files for images. Authors’ original file for figure 1 Authors’ original file for figure 2 Authors’ original file for figure 3

## References

[1] Tappy L, Le KA: Metabolic effects of fructose and the worldwide increase in obesity. Physiol Rev. 2010, 90: 23-46. 10.1152/physrev.00019.2009. 20086073 10.1152/physrev.00019.2009

[2] Stanley WC, Dabkowski ER, Hertel Ribeiro E, O’Connell KA: Dietary fat and heart failure: moving from lipotoxicity to lipoprotection. Circ Res. 2012, 110: 764-76. 10.1161/CIRCRESAHA.111.253104. 22383711 10.1161/CIRCRESAHA.111.253104 PMC3356700

[3] Mozaffarian D, Appel LJ, Van HL: Components of a cardioprotective diet: new insights. Circulation. 2011, 123: 2870-91. 10.1161/CIRCULATIONAHA.110.968735. 21690503 10.1161/CIRCULATIONAHA.110.968735 PMC6261290

[4] Mozaffarian D, Hao T, Rimm EB, Willett WC, Hu FB: Changes in diet and lifestyle and long-term weight gain in women and men. N Engl J Med. 2011, 364: 2392-404. 10.1056/NEJMoa1014296. 21696306 10.1056/NEJMoa1014296 PMC3151731

[5] Chess DJ, Stanley WC: Role of diet and fuel overabundance in the development and progression of heart failure. Cardiovasc Res. 2008, 79: 269-78. 10.1093/cvr/cvn074. 18343896 10.1093/cvr/cvn074

[6] Carpentier YA, Postois L, Malaisse WJ: n-3 fatty acids and the metabolic syndrome. Am J Clin Nutr. 2006, 83: 1499S-504. 16841860 10.1093/ajcn/83.6.1499S

[7] McLennan PL: Relative effects of dietary saturated, monounsaturated, and polyunsaturated fatty acids on cardiac arrhythmias in rats. Am J Clin Nutr. 1993, 57: 207-12. 8424390 10.1093/ajcn/57.2.207

[8] Hertel Ribeiro E, Fernandes AA, Meira EF, Batista PR, Siman FD, Vassallo DV: Soybean oil increases SERCA2a expression and left ventricular contractility in rats without change in arterial blood pressure. Lipids Health Dis. 2010, 9: 53-10.1186/1476-511X-9-53. 20504316 10.1186/1476-511X-9-53 PMC2894821

[9] Burniston JG: Adaptation of the rat cardiac proteome in response to intensity-controlled endurance exercise. Proteomics. 2009, 9: 106-15. 10.1002/pmic.200800268. 19053138 10.1002/pmic.200800268

[10] Laemmli UK: Cleavage of structural proteins during the assembly of the head of bacteriophage T4. Nature. 1970, 227: 680-5. 10.1038/227680a0. 5432063 10.1038/227680a0

[11] Neuhoff V, Arold N, Taube D, Ehrhardt W: Improved staining of proteins in polyacrylamide gels including isoelectric focusing gels with clear background at nanogram sensitivity using coomassie brilliant blue G-250 and R-250. Electrophoresis. 1988, 9: 255-62. 10.1002/elps.1150090603. 2466658 10.1002/elps.1150090603

[12] Vergote D, Bouchut A, Sautière PE, Roger E, Galinier R, Rognon A: Characterization of proteins differentially present in the plasma of biomphalariaglabrata susceptible or resistant to echinostomacaproni. Int J Parasitol. 2005, 35: 215-24. 10.1016/j.ijpara.2004.11.006. 15710442 10.1016/j.ijpara.2004.11.006

[13] Thomas PD, Campbell MJ, Kejariwal A, Mi H, Karlak B, Daverman R: PANTHER: a library of protein families and subfamilies indexed by function. Genome Res. 2003, 13: 2129-41. 10.1101/gr.772403. 12952881 10.1101/gr.772403 PMC403709

[14] Breslow JL: n-3 Fatty acids and cardiovascular disease. Am J Clin Nutr. 2006, 83 (suppl): 1477S-82. 16841857 10.1093/ajcn/83.6.1477S

[15] Barany K: ATPase activity of myosin correlated with speed of muscle shortening. J Gen Physiol. 1967, 5: 197-216. 10.1085/jgp.50.6.197. 10.1085/jgp.50.6.197 PMC2225740 4227924

[16] Weisser-Thomas J, Piacentino V, Gaughan JP, Margulies K, Houser SR: Calcium entry via Na/Ca exchange during the action potential directly contributes to contraction of failing human ventricular myocytes. Cardiovasc Res. 2003, 57: 974-85. 10.1016/S0008-6363(02)00732-0. 12650875 10.1016/s0008-6363(02)00732-0

[17] Corbett JM, Why HJ, Wheeler CH, Richardson PJ, Archard LC, Yacoub MH: Cardiac protein abnormalities in dilated cardiomyopathy detected by two-dimensional polyacrylamide gel electrophoresis. Electrophoresis. 1998, 19: 2031-42. 10.1002/elps.1150191123. 9740065 10.1002/elps.1150191123

[18] Van der Velden J, Papp Z, Boontje N, Zaremba R, de Jong JW, Janssen PML: The effect of myosin light chain 2 dephosphorylation on Ca^2+^ - ensitivity of force is enhanced in failing human hearts. Cardiovasc Res. 2003, 57: 505-14. 10.1016/S0008-6363(02)00662-4. 12566123 10.1016/s0008-6363(02)00662-4

[19] Heling A, Zimmermann R, Kostin S, Maeno Y, Hein S, Devaux B: Increased expression of cytoskeletal, linkage, and extracellular proteins in failing human myocardium. Circ Res. 2000, 86 (8): 846-53. 10.1161/01.RES.86.8.846. 10785506 10.1161/01.res.86.8.846

[20] Faber MJ, Dalinghaus M, Lankhuizen IM, Bezstarosti K, Dekkers DH, Duncker DJ: Proteomic changes in the pressure overloaded right ventricle after 6 weeks in young rats: correlations with the degree of hypertrophy. Proteomics. 2005, 5 (10): 2519-30. 10.1002/pmic.200401313. 15912512 10.1002/pmic.200401313

[21] Lam L, Tsoutsman T, Arthur J, Semsarian C: Differential protein expression profiling of myocardial tissue in a mouse model of hypertrophic cardiomyopathy. J Mol Cell Cardiol. 2010, 48: 1014-22. 10.1016/j.yjmcc.2009.08.015. 19715700 10.1016/j.yjmcc.2009.08.015

[22] Li C, Qiu Q, Wang Y, Li P, Xiao C, Wang H: Time course label-free quantitative analysis of cardiac muscles of rats after myocardial infarction. Mol BioSyst. 2014, 10: 505-10.1039/c3mb70422j. 24382414 10.1039/c3mb70422j

[23] Monreal G, Nicholson LM, Han B, Joshi MS, Phillips AB, Wold LE: Cytoskeletal remodeling of desmin is a more accurate measure of cardiac dysfunction than fibrosis or myocyte hypertrophy. Life Sci. 2008, 83: 786-94. 10.1016/j.lfs.2008.09.026. 18955067 10.1016/j.lfs.2008.09.026

[24] Pawlak A, Gil RJ, Walczak E, Seweryniak P: Desmin expression in human cardiomyocytes and selected clinical and echocardiographic parameters in patients with chronic heart failure. Kardiol Pol. 2009, 67 (9): 955-61. 19838951

[25] Gupta A, Akki A, Wang Y, Leppo MK, Chacko VP, Foster DB: Creatine kinase-mediated improvement of function in failing mouse hearts provides causal evidence the failing heart is energy starved. J Clin Invest. 2012, 122: 291-302. 10.1172/JCI57426. 22201686 10.1172/JCI57426 PMC3248286

[26] Spindler M, Meyer K, Stromer H, Leupold A, Boehm E, Wagner H: Creatine Kinase-deficient hearts exibit increased susceptibility to ischemia-reperfusion injury and impaired calcium homeostasis. Am J Physiol Heart Circ Physiol. 2004, 287 (3): 1039-45. 10.1152/ajpheart.01016.2003. 10.1152/ajpheart.01016.2003 15105171

[27] Crozatier B, Badoual T, Boehm E, Ennezat PV, Guenoun T, Su J: Role of creatine kinase in cardiac excitation-contraction coupling: studies in creatine kinase-deficient mice. FASEB J. 2002, 16: 653-60. 10.1096/fj.01-0652com. 11978729 10.1096/fj.01-0652com

[28] Momken I, Lechene P, Koulmann N, Fortin D, Mateo P, Doan BT: Impaired voluntary running capacity of creatine kinase-deficient mice. J Physiol. 2005, 565: 951-64. 10.1113/jphysiol.2005.086397. 15831533 10.1113/jphysiol.2005.086397 PMC1464549

[29] Marx JL: Oxygen free radicals linked to many diseases. Science. 1987, 235: 529-31. 10.1126/science.3810154. 3810154 10.1126/science.3810154

[30] Myers ML, Bolli R, Lekich RF, Hartley CJ, Roberts R: Enhancement of recovery of myocardial function by oxygen free-radical scavengers after reversible regional ischemia. Circulation. 1985, 72: 915-21. 10.1161/01.CIR.72.4.915. 4028384 10.1161/01.cir.72.4.915

[31] Wassell J: Haptoglobin: function and polymorphism. Clin Lab. 2000, 46 (11–12): 547-52. 11109501

[32] Haas B, Serchi T, Wagner DR, Gilson G, Planchon S, Renaut J: Proteomic analysis of plasma samples from patients with acute myocardial infarction identifies haptoglobin as a potential prognostic biomarker. J Proteome. 2011, 75: 229-36. 10.1016/j.jprot.2011.06.028. 10.1016/j.jprot.2011.06.028 21767674

[33] Tuncay E, Seymen AA, Tanriverdi E, Yaras N, Tandogan B, Ulusu NN: Gender related differential effects of omega-3 treatment on diabetes-induced left ventricular dysfunction. Mol Cell Biochem. 2007, 304 (1–2): 255-63. 10.1007/s11010-007-9508-4. 17530185 10.1007/s11010-007-9508-4

[34] Lemasters JJ, Holmuhamedov E: Voltage-dependent anion channel (VDAC) as mitochondrial governator–thinking outside the box. Biochim Biophys Acta. 2006, 1762 (2): 181-90. 10.1016/j.bbadis.2005.10.006. 16307870 10.1016/j.bbadis.2005.10.006

[35] Colombini M, Blachly-Dyson E, Forte M: VDAC, a channel in the outer mitochondrial membrane. Ion Channels. 1996, 4: 162-209. 10.1007/978-1-4899-1775-1_5 8744209

