# Supplementary material for: Cardiac protein changes in rats after soybean oil treatment: a proteomic study
Source: Lipids Health Dis. 2015 Apr 14;14:26. doi: 10.1186/s12944-015-0024-3 (PMC4446950; doi:10.1186/s12944-015-0024-3)

Supplementary material S1

2D-gels images obtained from four independent samples (4 biological replicates) per group (CT and TR).


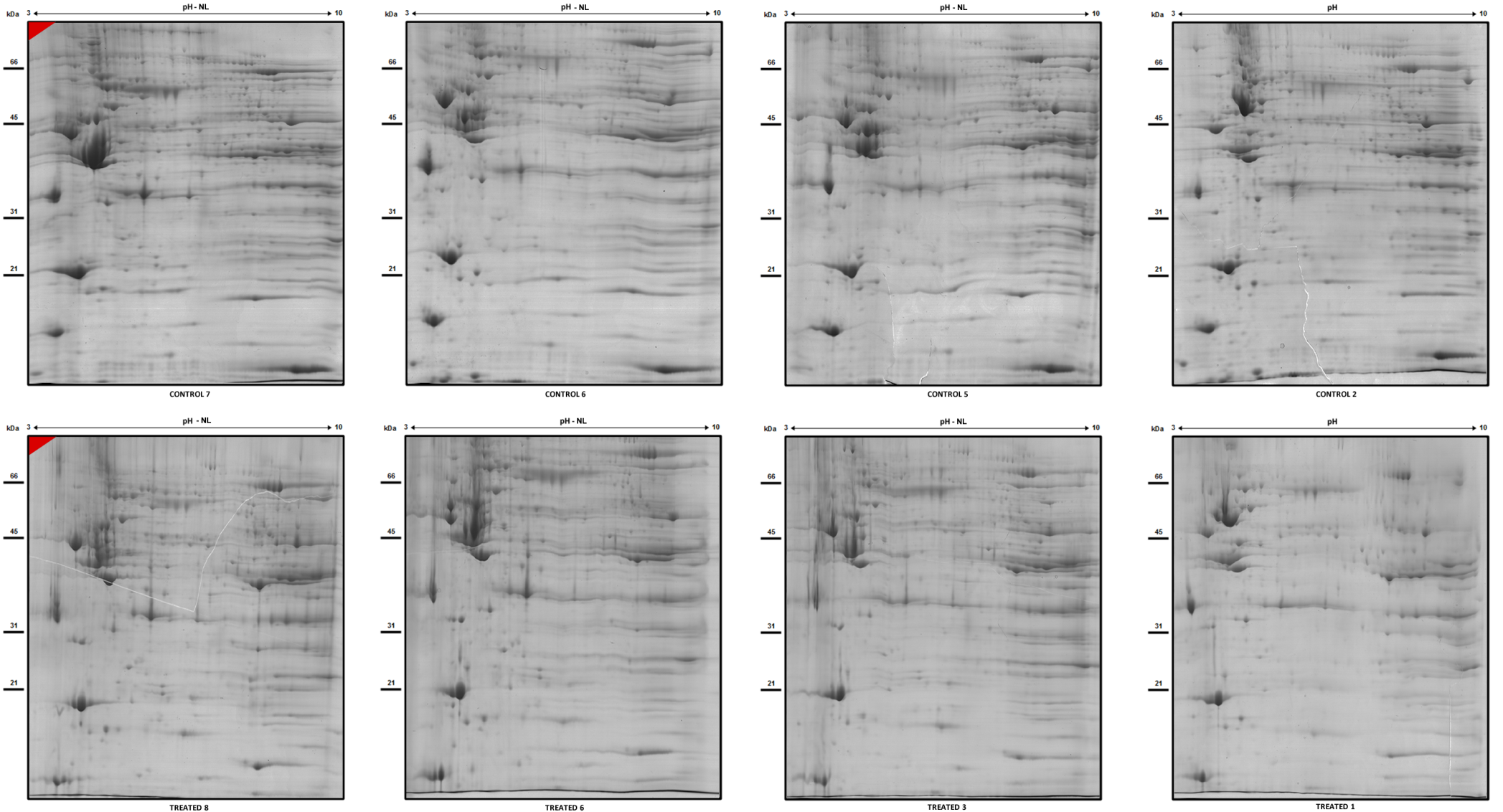

Supplement: Supplementary file 1 — Additional file 1:2-DE gels images obtained from four independent samples (4 biological replicates) per group (CT and TR).(DOC 2 MB) [file 12944_2015_24_MOESM1_ESM.doc]
