# Supplementary figures and images for: Cardiac protein changes in rats after soybean oil treatment: a proteomic study
Source: Lipids Health Dis. 2015 Apr 14;14:26. doi: 10.1186/s12944-015-0024-3 (PMC4446950; doi:10.1186/s12944-015-0024-3)

Supplementary material S2

Graphic of projection of the gels obtained by factor analysis.


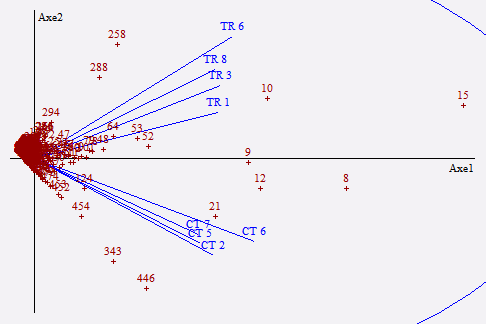

Supplement: Supplementary file 2 — Additional file 2:Graphic of projection of the gels obtained by factor analysis.(DOC 34 KB) [file 12944_2015_24_MOESM2_ESM.doc]

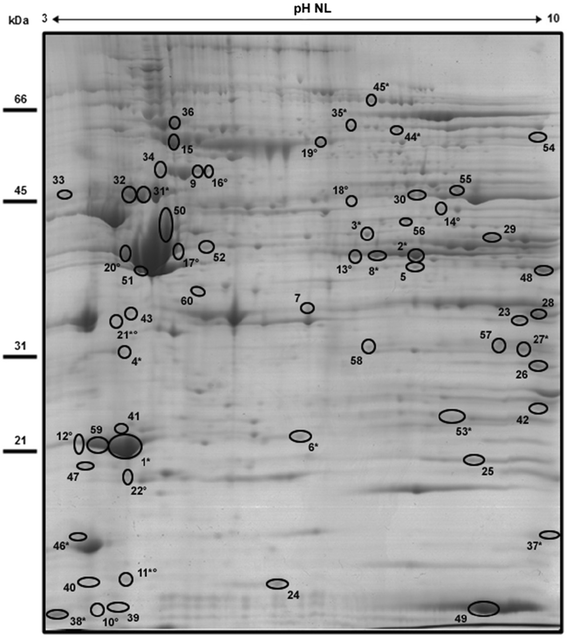

Supplement: Supplementary file 3 — Authors’ original file for figure 1 [file 12944_2015_24_MOESM3_ESM.gif]
