# Supplementary material for: Cardiac protein changes in rats after soybean oil treatment: a proteomic study
Source: Lipids Health Dis. 2015 Apr 14;14:26. doi: 10.1186/s12944-015-0024-3 (PMC4446950; doi:10.1186/s12944-015-0024-3)
Supplement: Supplementary file 4 — Authors’ original file for figure 2 [file 12944_2015_24_MOESM4_ESM.docx]

Table 1. Hemodynamic measures from control (CT) and soybean oil-treated rats (TR).

|  | **CT (n=8)** | **TR (n=8)** |
| --- | --- | --- |
| **HR,** bpm | 374 ± 18 | 342 ± 14 |
| **SBP,** mmHg | 114.9 ± 4.6 | 102.57 ± 4.88 |
| **DBP,** mmHg | 81.6 ± 5.9 | 73.75 ± 5.44 |
| **PAM,** mmHg | 98.8 ± 4.9 | 82.5 ± 6.08 |
| **LVSP,** mmHg | 128.4 ± 6.6 | 118.8 ± 5.07 |
| **LVEDP,** mmHg | 6.08 ± 0.41 | 4.31 ± 0.26* |
| **dP/dt_+,_** mmHg/s | 6656. ± 691 | 6066 ± 434 |
| **dP/dt _-,_** mmHg/s | 8061 ± 397 | 6994 ± 414 |

HR - heart rate; SBP - systolic blood pressure; DBP - diastolic blood pressure; LVSP left ventricle systolic pressure; LVEDP - left ventricle end diastolic pressure; dP/dt+ maximal rate of pressure development; dP/dt _-_  maximal rate of pressure decay. Data are mean ± S.E.M. Student "t" test, *p < 0.05 vs. CT
