# Supplementary material for: Cardiac protein changes in rats after soybean oil treatment: a proteomic study
Source: Lipids Health Dis. 2015 Apr 14;14:26. doi: 10.1186/s12944-015-0024-3 (PMC4446950; doi:10.1186/s12944-015-0024-3)
Supplement: Supplementary file 5 — Authors’ original file for figure 3 [file 12944_2015_24_MOESM5_ESM.docx]

Table 2. Proteins expressed differently in CT and TR groups

| **Spot^a^** | **Protein name (ID)** | **Variation of spot protein intensity in LV TR group** | **Biological process^b^** | **Identified peptide sequences** | **Mascot – score proteico^c^** | **pI (theoretical)** | **MM kDa (theoretical)** |
| --- | --- | --- | --- | --- | --- | --- | --- |
| ***UP-REGULATION*** | | | | | | | |
| **1** | Myosin light chain-3 (**P09542**) | ↑ | Muscle contraction | ITYGQCGDVLR | 96 | 5,03 | 22,2 |
| **2** | Creatine kinase M-type (**P00564)** | ↑ | [Phosphocreatine biosynthetic process](http://www.ebi.ac.uk/QuickGO/GTerm?id=GO:0046314) | GYTLPPHCSR;  DLFDPIIQDR; LGSSEVEQVQLVVDGVK; TGTAEMSSILEER;  FEEILTR;  GGDDLDPNYVLSSR; SFLVWVNEEDHLR; RGTGGVDTAAVGAVFDISNADR | 65  91  143  62  51  85  126  117 | 6,58 | 43,2 |
| **3** | Tu translation elongation factor, mitochondrial (predicted), isoform (**P49411**) | ↑ | [Protein biosynthesis](http://www.uniprot.org/keywords/KW-0648) | AEAGDNLGALVR;  YEEIDNAPEER; ADAVQDSEMVELVELEIR; KYEEIDNAPEER;  LLDAVDTYIPVPTR; GITINAAHVEYSTAAR; GEETPVIVGSALCALEQR; DLEKPFLLPVESVYSIPGR | 82  84  133  104  113  137  176  191 | 7,23 | 44,0 |
| **4** | Myosin light chain (**P09542**) | ↑ | Muscle contraction | HVLATLGER; NKDTGTYEDFVEGLR; AAPAPAAAPAAAPEPERPK; HVLATLGER;  NKDTGTYEDFVEGLR; AAPAPAAAPAAAPEPERPK | 72  124  130  72  124  130 | 5,03 | 22,2 |
| **21** | Preprohaptoglobin (**P06866**) | ↑ | [Acute phase](http://www.uniprot.org/keywords/KW-0011) [Immunity](http://www.uniprot.org/keywords/KW-0391) | GSFPWQAK;  MGYVSGWGR;  YVMLPVADQEK;  SCAVAEYGVYVR | 43  52  48  64 | 6,10^d^ | 30,4 |
| ***DOWN-REGULATION*** | | | | | | | |
| **6** | Thioredoxin-dependent peroxide reductase (**Q9Z0V6**) | ↓ | Antioxidant | SVEETLR | 47 | 7,15 | 28,3 |
| **8** | Creatine kinase M-type (**P00564**) | ↓ | Phosphocreatine biosynthetic process | GTGGVDTAAVGAVFDISNADR | 231 | 6,58 | 43,2 |
| **26** | Vdac 1 protein, partial (**Q9Z2L0**) | ↓ | [Apoptosis](http://www.uniprot.org/keywords/KW-0053)/ [Ion transport](http://www.uniprot.org/keywords/KW-0406) | GYGFGLIK;  LETAVNLAWTAGNSNTR; KLETAVNLAWTAGNSNTR; VNNSSLIGLGYTQTLKPGIK; EHINLGCDVDFDIAGPSIR; WNTDNTLGTEITVEDQLAR; TDEFQLHTNVNDGTEFGGSIYQK | 72  98  167  143  207  189  217 | 8,55 | 30,8 |
| **31** | Desmin (**P48675**) | ↓ | [Muscle protein](http://www.uniprot.org/keywords/KW-0514) | LQEEIQLR;  RIESLNEEIAFLK | 65  66 | 5,21 | 53,4 |
| **37** | NADH desidrogenase [ubiquinone] (**Q91VD9**) | ↓ | [Electron transport](http://www.uniprot.org/keywords/KW-0249)/ [Respiratory chain](http://www.uniprot.org/keywords/KW-0679) | HGWSYDVEGR;  SYGANFSWNKR; LDVTPLTGVPEEHIK | 90  33  82 | 10,14 | 19,7 |
| **44** | Long-chain-fatty-acid-CoA ligase 1 (**P18163**) | ↓ | Fatty acid metabolism | VLKPTIFPVVPR | 56 | 5,67 | 76,3 |
| **45** | Glycogen phosphorylase, muscle form (**P09812**) | ↓ | Carbohydrate metabolic process | DYYFALAHTVR | 98 | 6,91 | 97,7 |
| **46** | chaperone activity of bc1 complex-like, mitochondrial (**Q5BJQ0**) | ↓ | [Ubiquinone biosynthesis](http://www.uniprot.org/keywords/KW-0831) | EAGLSGQATSPLGR; EGPAPAYVSSGPFR | 109  130 | 98 | 72,7 |
| **53** | ES1 protein homolog, mitochondrial precursor (**P56571**) | ↓ |  | ITNLAQLSAANHDAAIFPGGFGAAK | 98 | 9,11 | 28,4 |

^a^ Spot ID - The numbers correspond to the specific spots as indicated in Figure 1.^b^Functional categories according to Gene Ontology, Panther and UniProt biological process annotations. ^c^ With statistical significance (p <0.05) in protein homology and identity.^*^pI related to the protein, it has not been found the pI of prepropeptide.
